# Temporal Changes in Prosaposin Expression in the Rat Dentate Gyrus after Birth

**DOI:** 10.1371/journal.pone.0095883

**Published:** 2014-05-28

**Authors:** Midori Morishita, Hiroaki Nabeka, Tetsuya Shimokawa, Kyojy Miyawaki, Takuya Doihara, Shouichiro Saito, Naoto Kobayashi, Seiji Matsuda

**Affiliations:** 1 Department of Anatomy and Embryology, Ehime University Graduate School of Medicine, Toon, Ehime, Japan; 2 Shikokuchuoh Medical Welfare General College, Shikokuchuoh, Ehime, Japan; 3 Laboratory of Veterinary Anatomy, Faculty of Applied Biological Sciences, Gifu University, Yanagido, Gifu, Japan; 4 Medical Education Center, Ehime University Graduate School of Medicine, Toon, Ehime, Japan; Nathan Kline Institute and New York University School of Medicine, United States of America

## Abstract

Neurogenesis in the hippocampal dentate gyrus occurs constitutively throughout postnatal life. Adult neurogenesis includes a multistep process that ends with the formation of a postmitotic and functionally integrated new neuron. During adult neurogenesis, various markers are expressed, including GFAP, nestin, Pax6, polysialic acid-neural cell adhesion molecule (PSA-NCAM), neuronal nuclei (NeuN), doublecortin, TUC-4, Tuj-1, and calretinin. Prosaposin is the precursor of saposins A–D; it is found in various organs and can be excreted. Strong prosaposin expression has been demonstrated in the developing brain including the hippocampus, and its neurotrophic activity has been proposed. This study investigated changes in prosaposin in the dentate gyrus of young and adult rats using double immunohistochemistry with antibodies to prosaposin, PSA-NCAM, and NeuN. Prosaposin immunoreactivity was intense in the dentate gyrus at postnatal day 3 (P3) and P7, but decreased gradually after P14. In the dentate gyrus at P28, immature PSA-NCAM-positive neurons localized exclusively in the subgranular zone were prosaposin-negative, whereas mature Neu-N-positive neurons were positive for prosaposin. Furthermore, these prosaposin-negative immature neurons were saposin B-positive, suggesting that the neurons take up and degrade prosaposin. *In situ* hybridization assays showed that prosaposin in the adult dentate gyrus is dominantly the Pro+9 type, a secreted type of prosaposin. These results imply that prosaposin secreted from mature neurons stimulates proliferation and maturation of immature neurons in the dentate gyrus.

## Introduction

Prosaposin (PS) is the precursor of saposins A–D and is predominantly expressed in the brain, muscles [33]–[35], lymphatic tissue [39], and other organs [4], [43], [45]. It is also found in various excretions such as cerebrospinal fluid [15]. PS is necessary for sphingolipid hydrolysis in lysosomes [29] and also acts as an extracellular protein [10], [15]. PS has been reported to facilitate sciatic nerve regeneration [21] and ameliorate cavity formation following stab woud injury of the cortex [19]. Neurotrophic activity of PS is attributed to a 12-amino-acid stretch located at the N-terminal part of saposin C [30], [32]. PS an PS-derived peptides prevent cell death in cerebellar granuleneurons [44], hippocampal neurons [27], [35] and dopaminergic neurons [12], [24]. PS has been suggested to have neurotrophic activity from a neuropathololoic study of human PS deficiency [41].

PS has two isoforms, Pro+9 and Pro+0, caused by differential splicing at the saposin B domain [17]. Pro+9 has a 9-base insertion whereas Pro+0 does not. Pro+0 is mainly transported into lysosomes, and Pro+9 is predominantly secreted out of a cell [25]. Although Pro+9 expression in the brain increases during embryonic development [5], its exact role remains unknown.

In general, neurons stop proliferating in adulthood. In the hippocampal dentate gyrus and the olfactory bulb in adult mammals, however, neurogenesis lasts throughout life [1], [9], [31], [37]. The rate of neurogenesis in the dentate gyrus changes with various physiological or pathological situations [22]. Adult neurogenesis includes a multistep process (proliferation, differentiation, migration, targeting, and synaptic integration) that ends with the formation of a postmitotic, functionally integrated new neuron [46]. In mammals, 85% of granule cells are generated after birth [2]. In particular, in rats, granule cells are formed almost completely within the first 3 postnatal weeks [36]. Hippocampal neurogenesis may be related to memory and learning [8], [40].

Neuronal precursor cells are distributed throughout the hilus of the dentate gyrus in the early stages after birth but gradually locate to the subgranular zone (SGZ) facing the hilus where they differentiate as they grow [2]. During differentiation, various markers, such as GFAP, nestin, Pax6, polysialic acid-neural cell adhesion molecule (PSA-NCAM), neuronal nuclei (NeuN), doublecortin, TUC-4, Tuj-1, and calretinin, are expressed [38]. PSA-NCAM is expressed in immature neuronal cells and enables identification of young cells after cell division [47]. However, NeuN is expressed in mature neurons [28].

The dentate gyrus in the hippocampus, which plays a crucial role in memory formation, is one of two brain regions in which neurogenesis occurs even in adulthood [11], [13], [20], [22], [50]. Neurogenesis in the adult hippocampus is regulated by several growth factors, including brain-derived neurotrophic factor (BDNF), never growth factor (NGF), neurotrophin-3 (NT3), insulin-like growth factor (IGF) and vascular endothelial growth factor (VEGF). In a previous report, we showed that prosaposin (PS) and PS-related peptide promoted the survival and neurite outgrowth of cultured hippocampal neurons and protected against ischemia-induced learning disability and hippocampal neuronal loss [21], [29]. Characterization of PS expression patterns in the hippocampal dentate gyrus may lead to further elucidation of the role of PS in the postnatal brain [52]. In this study, we performed multiple immunofluorescence analyses in the dentate gyrus of young rats using the neuronal differentiation markers PSA-MCAM and NeuN, as well as PS, to clarify the role of PS in development. The antibody against PS used in this study recognizes PS but not monomers of saposin and therefore shows exact PS localization. In addition, we performed *in situ* hybridization assays of Pro+0 and Pro+9 to investigate PS production in the dentate gyrus.

## Materials and Methods

The protocol for this trial and supporting ARRIVE checklist are available as supporting information; see Checklist S1.

### Anti-rat prosaposin antibody (anti-PS) and Western blotting

A specific polyclonal antibody against PS was generated by immunizing rabbits with a synthetic oligopeptide corresponding to the proteolytic portion of rat PS (M19936; Collard et al.) (409-PKEPAPPKQPEEPKQSALRAHVPPQK-434) [6]. The oligopeptide was selected from the sequence between saposin C and saposin D, and does not encode any saposins. The antiserum was affinity purified with the oligopeptide. All procedures were performed by Medical and Biological Laboratories Co., Ltd. (Nakaku, Nagoya, Japan). The immunoblot procedure was performed as described previously [39]. Total protein extracts from the hippocampi of male Wistar rats at P7 or P28 were heated at 60°C for 10 min and pelleted by centrifugation at 12,000× *g*. The heat-stable fraction was separated by sodium dodecyl sulfate-polyacrylamide gel electrophoresis and transferred to polyvinylidine difluoride membranes. After blocking, the membrane was immunolabeled with rabbit anti-saposin B, D, and PS. Immunoreactions were visualized using horseradish peroxidase-conjugated anti-rabbit immunoglobulin and enhanced chemiluminescence (ImmunoStar; Wako, Osaka, Japan). Immunoreactive protein bands were visualized with an LAS-4000 luminescence image analyzer (GE Healthcare, Tokyo, Japan). The specificity of the anti-saposin B and D antiserum has been previously reported using Western blotting [18].

### Tissue preparation for immunochemistry

Male Wistar rats aged P3, P7, P14, and P28 were used in group of six. All animals were housed at a constant temperature (22°C) under a 12∶12-h light–dark cycle and given food and water ad libitum. The following experiments were conducted in accordance with the Guide for Animal Experimentation at Ehime University School of Medicine, Japan. The current study, in addition to its protocol, was approved by the Committee on the Ethics of Animal Experiments of the Ehime University School of Medicine (Permit Number: A21-2). All surgery was performed under sodium pentobarbital anesthesia, and all efforts were made to minimize suffering. After anesthetizing by intraperitoneal injection of chloral hydrate (10 mg/kg), animals were transcardially perfused with heparinized phosphate-buffered saline (PBS), followed by 4% paraformaldehyde in 0.1 M phosphate buffer (PB; pH 7.4). Brains were removed and post-fixed overnight in the same fixative. They were immersed in 0.1 M PB containing 30% sucrose and frozen on dry ice. Coronary sections (5 µm thick) were cut using a cryostat, thaw-mounted onto silane-coated slides, and stored at −80°C until use.

### Triple-immunofluorescence staining

The anti-PS antibody was used to evaluate PS. As noted above, this antibody recognizes the intermediate sequence between saposin C and saposin D, and does not react with saposin A, B, C, or D. Its use as a specific antibody was demonstrated by immunoblotting. NeuN and PSA-NCAM were used as markers to distinguish immature or mature granule cells in the dentate gyrus. NeuN is globally used as a marker of mature neurons that are past cell division, whereas PSA-NCAM is useful as a marker of relatively immature neurons with axons and dendrites in the postmitotic differentiation step. Localizations of PS, NeuN, and PSA-NCAM were examined using these antibodies, and the relationship to neurogenesis was evaluated.

Cryosections were air-dried and washed for 15 min in PBS plus Tween 20 (PBS-T). Sections were blocked overnight in PBS (0.1 M) containing 5% bovine serum albumin, 5% normal swine serum, and 0.1% NaN_3_, followed by incubation with the primary antibodies: rabbit polyclonal anti-PS IgG (1∶250), mouse monoclonal anti-PSA-NCAM IgM (1∶1000), or mouse monoclonal anti-NeuN IgG (1∶1000). Sections were washed twice for 15 min in PBS and reacted with the secondary antibodies [Cy3 anti-rabbit IgG (1∶500) and FITC anti-mouse IgG and IgM (1∶300)]. DAPI was added for 2 h to counterstain the nuclei. Finally, sections were washed twice for 15 min in PBS, mounted using Permafluor Aqueous Mounting media (Thermo Shandon; Thermo Scientific, West Palm Beach, FL, USA), and observed using fluorescence microscopy (Biozero; Keyence, Tokyo, Japan).

### Laser microscope observations

Laser microscopy was used to visualize the cellular localization patterns of PS using immunofluorescence. Sections (1-µm thickness) from the dentate gyrus of postnatal day (P) 28 rats were triple-stained with antibodies against polysialylated-neural cell adhesion (PSA-NCAM), saposin B and 4′,6-diamidino-2-phenylindole (DAPI) and observed using laser microscopy. Additionally, 1-µm sections from rats at P1, P7, P14 and P21 were triple-stained with antibodies against PS, microtubule-associated protein (Map-II) and DAPI, and assessed using laser microscopy. High-resolution confocal images were taken using an A1 confocal microscope equipped with a 60× oil immersion lens (numerical aperture 0.95; Nikon, Tokyo, Japan). Single-plane 1024×1024 images were captured using a 33.4 µm pinhole with 3× zoom.

### In situ hybridization

Brains were removed, frozen on dry ice, and cut into 10-µm sections using a cryostat for *in situ* hybridization. Three antisense 36-mer oligonucleotide probes AS1, AS3, and AS4, and one sense probe S1, were commercially synthesized (Operon Biotechnologies, Huntsville, AL, USA). AS1 was complementary to bases 1704–1739 in the 3′-untranslated region of the PS cDNA, AS3 contained the 9-base insertion to detect Pro+9 mRNA, and AS4 excluded the 9-base insertion and so it detected Pro+0 mRNA. For the control, the sense probe S1 was complementary to AS1. The sequences of the four probes are as follows:

•AS1: 5′- TTCATTACCCTAGACCCACAAGTAGGCGACTTCTGC-3′

•S1: 5′- GCAGAAGTCGCCTACTTGTGGGTCTAGGGTAATGAA-3′

•AS3: 5′-CTTGGGTTGCTGATCCTGCATGTGCATCATCATCTG-3′

•AS4: 5′-TTCCTTGGGTTGCATGTGCATCATCATCTGGACGGC-3′

Frozen sections were fixed for 15 min in 4% paraformaldehyde in 0.1 M PBS (pH 7.4), rinsed with standard 4× saline citrate (SSC, pH 7.4), and dehydrated through a graded ethanol series. Sections were hybridized overnight at 41°C with ^35^S-labeled antisense or sense probes (1.0×10^7^ cpm/ml) in hybridization buffer (50% formamide, 1% Denhardt's solution, 250 µg/ml tRNA, 0.1% g/ml dextran sulfate, 0.2 M PB, and 0.02 mM DTT in 4× SSC). After hybridization, sections were rinsed three times with 1× SSC at 55°C for 20 min, dehydrated through a graded ethanol series, coated with NBT2 emulsion (Eastman Kodak, Rochester, NY, USA), and exposed for 3 weeks at 4°C. Finally, sections were developed using D-19 developer (Eastman Kodak) and observed under a dark-field microscope.

### Statistical analyses of digital images

We obtained digital images of the PS hybridization signals in the dentate gyrus sections, as well as images after *in situ* hybridization without hematoxylin and eosin (HE) counterstaining. The average gray value of all pixels in each image was determined using the NIH 1.56 software (public domain software by Dr. Steve Barrett). The statistical significance of the values was examined by one-way analysis of variance (ANOVA) and Scheffe's test using the StatView software (Abacus Concepts Inc., Berkeley, CA). *P*-values <0.05 were considered to indicate statistical significance.

## Results

### Western blotting

**Figure 1:**
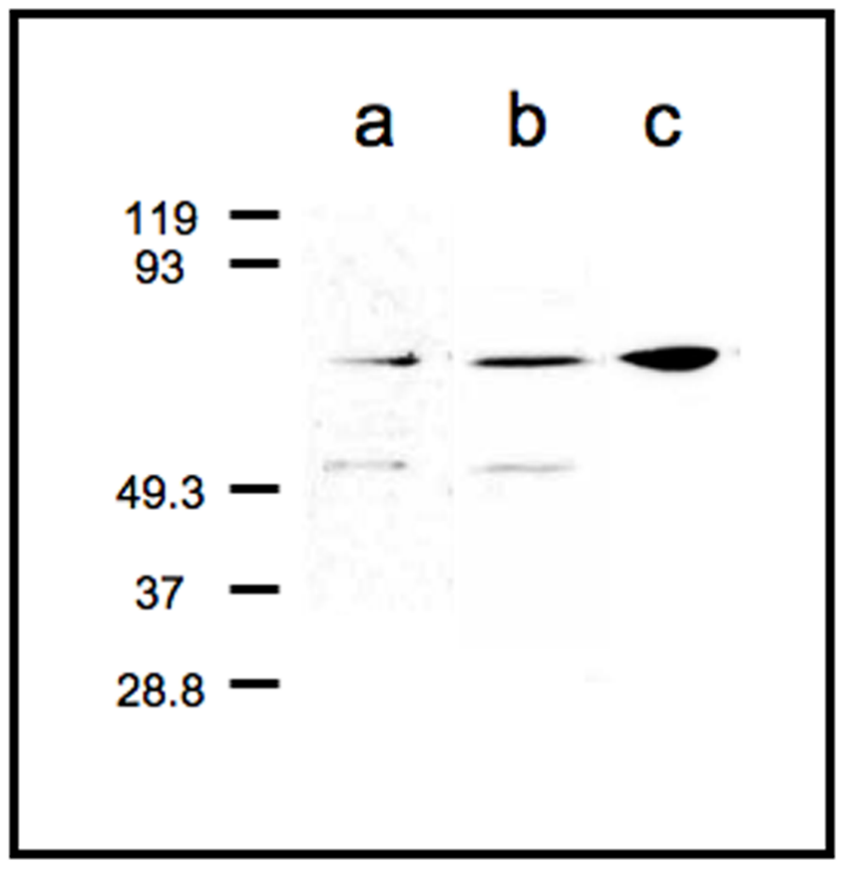
Western blot of hippocampal protein extracts. Stained with anti-saposin B (*a*) and D (*b*) antibodies. Two bands of approximately 50 kDa and 69 kDa (PS) were detected. (*c*) Stained with the specific anti-PS antibody. Only one band of PS was detected.

To examine the specificity of the rabbit anti-PS antibody, its reactivity was compared with the specificity of rabbit anti-saposin B and D antibodies using immunoblotting. Two bands were detected upon immunoblotting with the anti-saposin B and D antibodies (Fig. 1*a,b*). These bands were identified using markers, and the upper band was approximately 69 kDa, the molecular size of PS. These results agree with a previous report [18], [39]. The faint lower band was approximately 50 kDa and likely trisaposin. In contrast, only a single band was observed upon immunoblotting with the anti-PS antibody (Fig. 1*c*) and was identified as PS.

Our immunoblotting results show that the rabbit anti-PS antibody used in this study specifically recognizes PS and does not recognize di- or trisaposin. Therefore, the specificity of the anti-PS antibody was demonstrated.

### PS and PSA-NCAM triple-immunofluorescence staining

**Figure 2:**
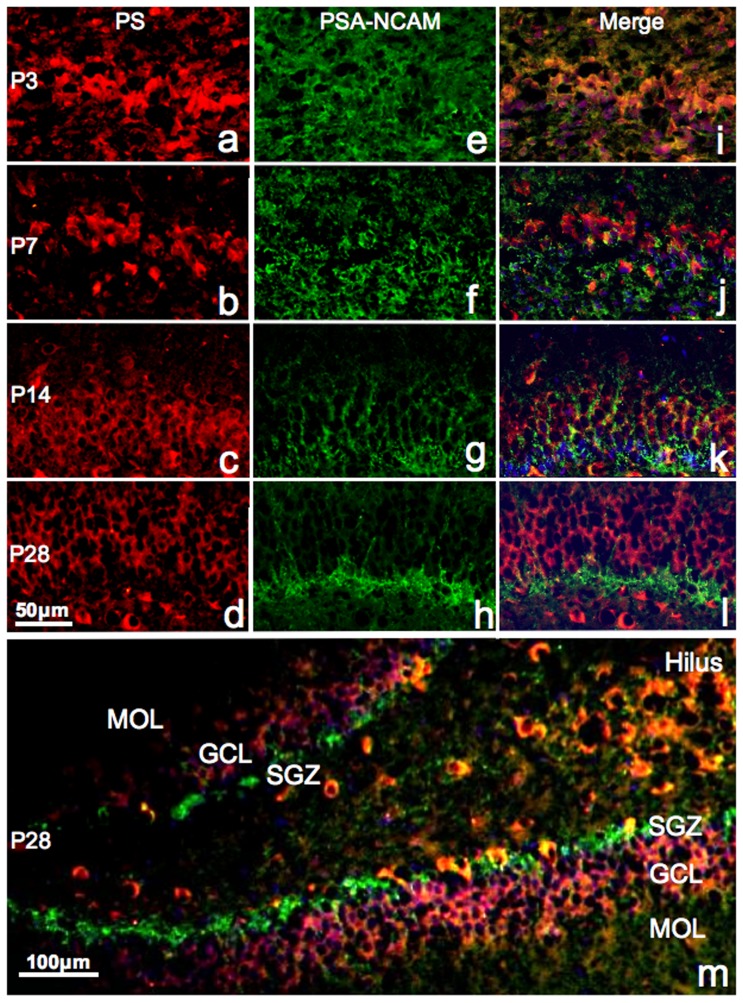
Fluorescent immunoreactivity of PS and PSA-NCAM. Fluorescent micrographs of the upper blade of the dentate gyrus showing immunoreactivity of PS (red, *a–d *), PSA-NCAM (green, *e–h*), and their merge (*i–l *) at P3 (*a*,*e*,*i*), P7 (*b*,*f*,*j *), P14 (*c*,*g*,*k*), and P28 (*d*,*h*,*l *). Nuclei are stained with DAPI (blue). Note that some double-positive cells were observed at P14 (*k*), and prosaposin-negative and PSA-NCAM-positive neurons clearly localized in the SGZ at P28 (*d*,*h*,*l *). A low power image of dentate gyrus double-stained with PSA-NCAM and PS at P28 is shown in panel *m*. MOL: molecular layer, GCL: ganglion cell layer, SGZ: subgranular zone.

In neurons from the dentate gyrus at P3 and P7, we observed stronger PS immunoreactivity at P14 (Fig. 2*a–d*). Immunoreactivity was observed both in the cytoplasm and nuclei at P3 (Fig. 2*a*) and P7 (Fig. 2*b*), but was predominantly cytoplasmic after P14 (Fig. 2*c,d*). The immunoreactivities decreased after P14 because the nuclei were enlarged and the cytoplasm was relatively decreased (Fig. 2*c,d*). PS-single positive cells were observed in the outer granule cell layer and CA4 region, but not in the SGZ at P28 (Fig. 2*d,l*).

PSA-NCAM immunoreactivity was clearly observed in the cytoplasm and dendrites (Fig. 2*e–h*). The staining was diffuse throughout all layers of the dentate gyrus at P3 (Fig. 2*e*), but was localized to the SGZ after P7 (Fig. 2*f–h*). These data indicate that neuronal proliferation actively occurs in all layers of the dentate gyrus at P3, but is restricted to the SGZ in adult animals.

Based on the localization of PS-single positive cells in the outer granule cell layer (Fig. 2*d*) and PSA-NCAM-single positive cells in the SGZ (Fig. 2*h*), the single positive cells showed completely complementary distribution (Fig. 2*l,m*).

A few double-positive cells were observed at P7 (Fig. 2*j*) and some were also found at P14 (Fig. 2*k*). However, double-positive cells were rarely detected at P28 (Fig. 2*l*). Figure 2*k and l* show PSA-NCAM-positive cells in the SGZ extending their dendrites to the granule cell layer with PS-positive cells. A low power image of the dentate gyrus double-stained with PSA-NCAM and PS at P28 is shown in Figure 2*m*.

### PS and NeuN triple immunofluorescence

**Figure 3:**
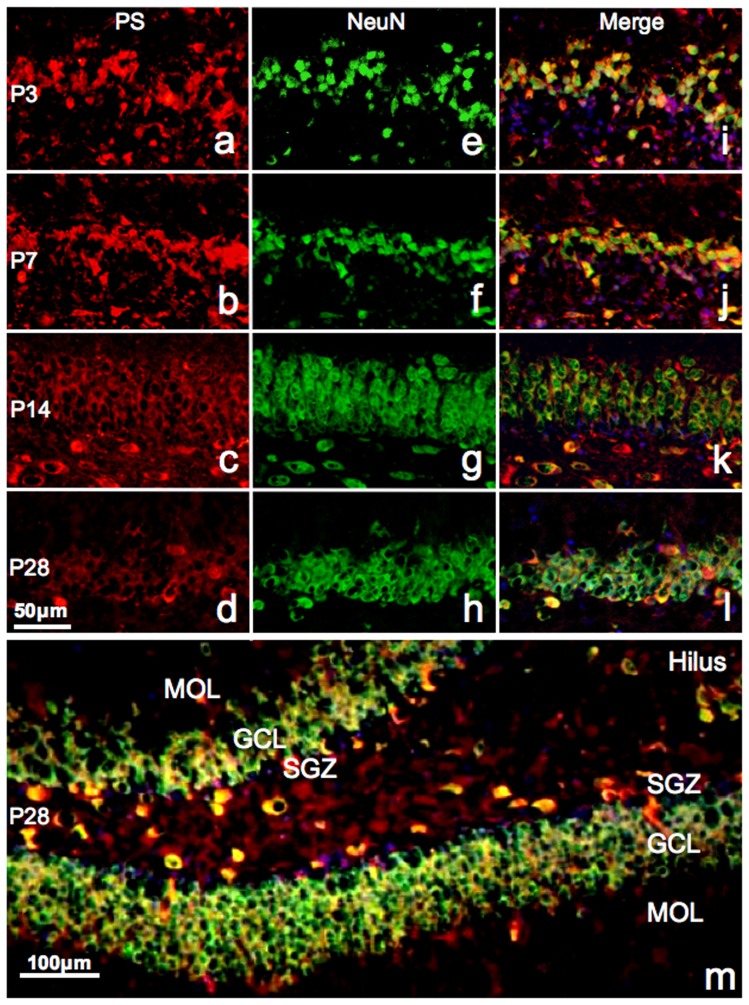
Fluorescent immunoreactivity of PS and NeuN. Fluorescent micrographs of the upper blade of the dentate gyrus showing immunoreactivity of PS (red, *a–d* ), NeuN (green, *e-h*), and their merge (*i–l* ) at P3 (*a, e, i* ), P7 (*b, f, j* ), P14 (*c, g, k*), and P28 (*d, h ,l* ). Nuclei are stained with DAPI (blue). A low-power image of the dentate gyrus double-stained with NeuN and PS at P28 is shown in panel *m*. Note that the majority of prosaposin-positive neurons are also NeuN-positive at all stages.

The results of prosaposin immunofluorescence (Fig. 3*a–d*) were similar to those in Figure 2*a–d*; however, its localization in the nuclei was more prominent at P3 (Fig. 3*a,e,i*). NeuN expresses in differentiated neurons, and NeuN immunoreactivity was observed in both the nucleus and cytoplasm. The granule cell layers at P3 and P7 were thinner (Fig. 3*e,f*) than at P14 (Fig. 3*g,h*).

Double-positive cells were widely observed in all sections. Only DAPI-positive, PS-, and NeuN-double negative cells were observed in the SGZ (Fig. 3*i–l*). A low power image of the dentate gyrus double-stained with NeuN and PS at P28 is shown in Figure 3*m*.

### Saposin B and PSA-NCAM double immunofluorescence

**Figure 4:**
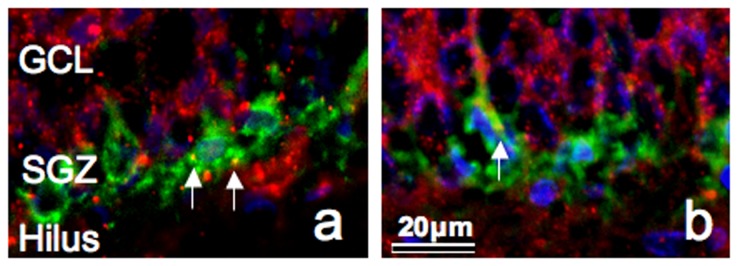
Fluorescent immunoreactivity of saposin B and PSA-NCAM. Light micrographs of the SGZ in the dentate gyrus at P28 stained with saposin B (red), PSA-NCAM (green) and DAPI (blue). Note that saposin B immunoreactivity (arrows) is frequently detected in PSA-NCAM-positive cells, where PS immunoreactivity is rarely observed.

Saposin B immunoreactivity is frequently detected in PSA-NCAM-positive cells in the SGZ where PS immunoreactivity is rarely observed (Fig. 4*a,b*). The double staining is not caused by overlapping cells but rather the co-localization of saposin B and PSA-NCAM in one cell because these photographs were taken using a confocal microscope with a focal depth of 1 µm.

### Immunoreactivity of PS in the nuclei and cytoplasm

**Figure 5:**
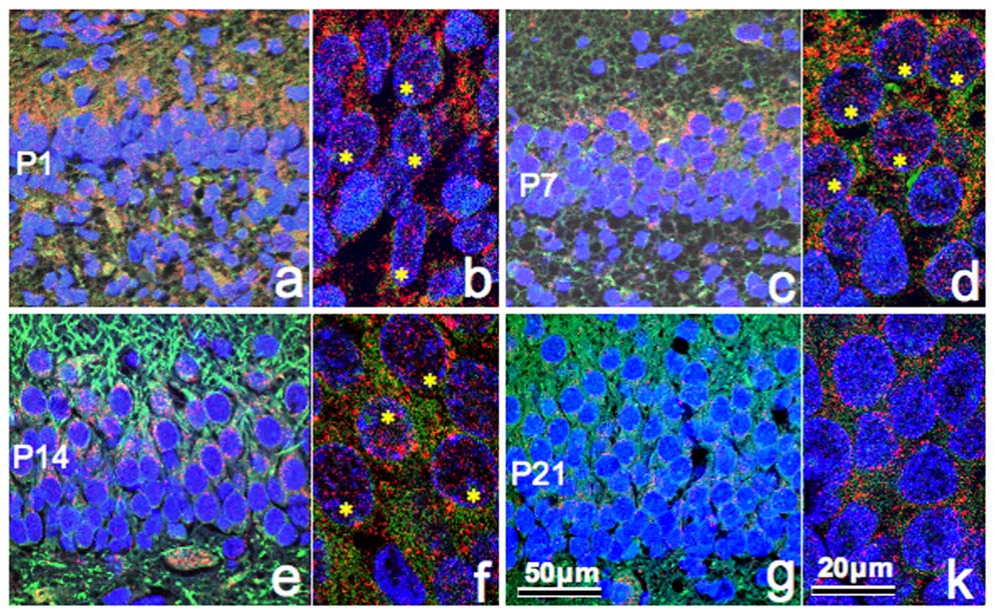
Fluorescent immunoreactivity of prosaposin (PS), microtubule-associated protein (Map-II) and 4′,6-diamidino-2-phenylindole (DAPI). Fluorescent micrographs of the upper blade of the dentate gyrus show immunoreactivity of PS (red), Map-II (green) and DAPI (blue) at postnatal day (P)1 (*a*, *b*), P7 (*c, d *), P14 (*e, f *) and P21 (*g, k*). Note that a number of nuclei (asterisks) at P1, P7 and P14 contain PS immunoreactivity, but those at P21 contain only background levels of immunoreactivity (*k*).

Laser microscopy images at higher magnification (Fig. 5) were examined for PS immunoreactivity in the nuclei and cytoplasm at P1, P7, P14 and P21. The results showed PS localization in the apical cytoplasm of granule cells at all stages and in the nuclei at P1, P7 and P14, but not at p21. PS immunoreactivity in the nuclei was found mainly in the dispersed chromatin (euchromatin), but not in the condensed chromatin (heterochromatin) at all stages (Fig. 5).

### *In situ* hybridization assays

**Figure 6:**
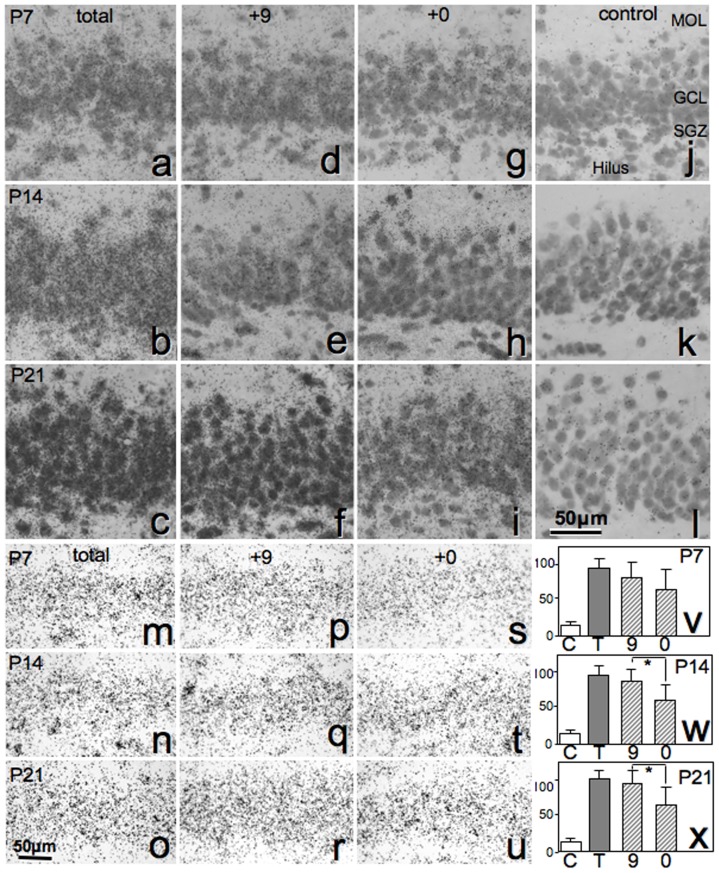
Developmental changes in Pro+0 and Pro+9 mRNA levels. Expression of total (Pro+0 and Pro+9) mRNA (*a–c*, *m–o,* using probe PS-AS1), Pro+0 mRNA (*d–f*, *p–r,* using probe PS-AS4), Pro+9 mRNA (*g–i, s–u,* using probe PS-AS3) and negative control (*j–l,* using probe S1) in the dentate gyrus at P7 (*a*, *d*, *g, j, m, p, s, v*), P14 (*b*, *e*, *h, k, n, q, t*) and P21 (*c*, *f*, *I, l, o, r, u, x*). Cells depicted in *a–l* were counterstained with hematoxylin and eosin (HE). At all stages, Pro+9 mRNA staining was of greater intensity than Pro+0 mRNA. In *v–x*, the columns indicate control (C), total (T), Pro+9 mRNA (9) and Pro+0 mRNA (0). *P<0.05.

**Figure 7:**
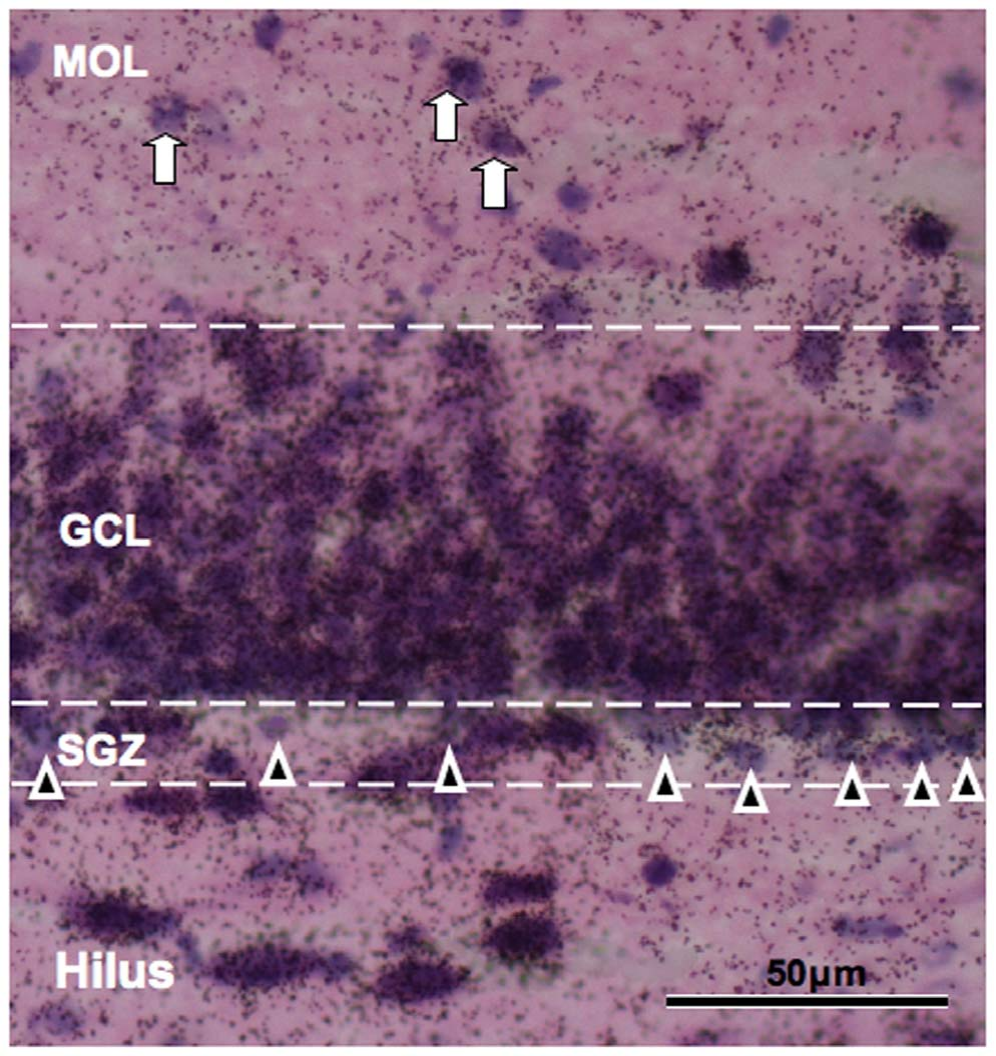
*Expression of* Pro+9 mRNA at P21. Pro+9 mRNA expression in the dentate gyrus at P21. Neurons (arrowheads) in the subgranular zone (SGZ) show lower staining levels of Pro+9 mRNA expression compared with neurons in the granule cell layer (GCL). Neurons in the hilus show intense staining of Pro+9 mRNA expression. A number of neurons (arrows) in the molecular layer (MOL) also show moderate mRNA expression levels.

Pro+0, Pro+9 and total PS expression levels in the dentate gyrus were assessed in P7, P14 and P21 animals. HE counterstaining revealed that the dentate gyrus thickness increased with developmental stage progression in rat (Fig. 6 *a–l *). As comparison of image analyses among the stages was difficult, we compared the hybridization signal intensities among the sections in the same stage treated with different probes. All sections without HE counterstaining (Fig. 6 *m–u*) showed greater expression of Pro+9 mRNA than Pro+0 mRNA (Fig. 6 *v–x*). Higher-magnification images with HE counterstaining revealed that neurons in the subgranular zone (SGZ) showed weak Pro+9 expression at P21, in agreement with PS immunoreactivity. The highest Pro+9 mRNA expression was observed in CA4 neurons in the hilus, and moderate signals were observed in a number of interneurons in the molecular layer (Fig. 7).

## Discussion

This study investigated PS expression in mature and immature hippocampal granule cells using double immunohistochemistry using antibodies against PS and PSA-NCAM (a marker of immature neurons in the dentate gyrus) or against PS and NeuN (a marker of mature neurons).

Furthermore, we analyzed the expression of the secretory type (Pro+9) or lysosomal type (Pro+0) of PS mRNA in the developing dentate gyrus from P3 to P21.

**Figure 8:**
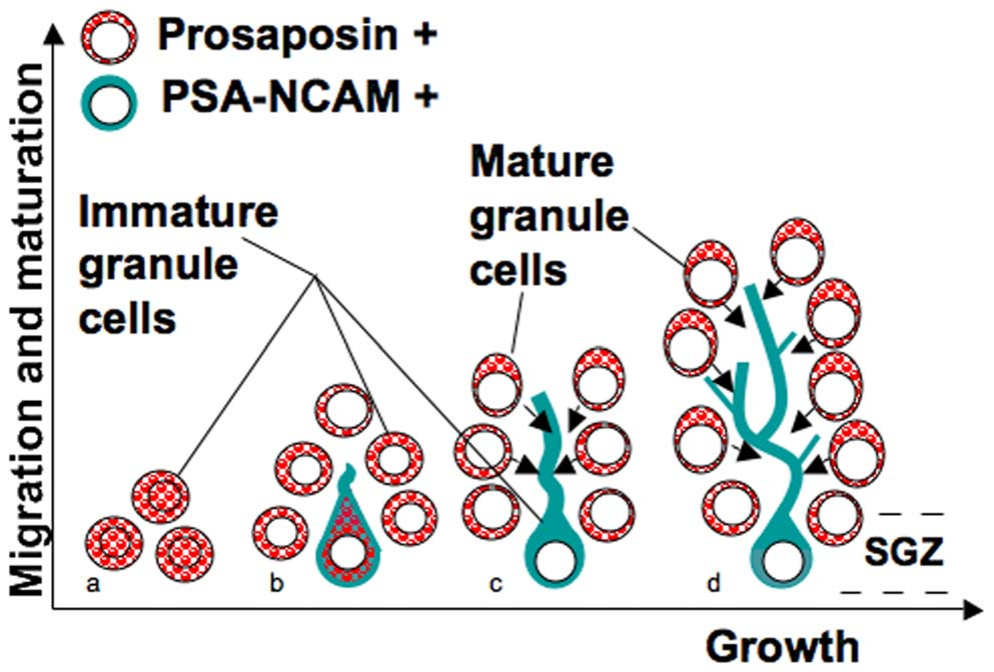
A scheme showing PS expression. A scheme showing the stages of neurogenesis and the expression pattern of PS and PSA-NCAM in the dentate gyrus at P3 (*a*), P7 (*b*), P14 (*c*), and P28 (*d*). Arrows indicate putative paracrine secretion of PS.

To illustrate our results, the development of PS-positive neurons at each stage is summarized in a scheme shown in Figure 8*a–d*. PS immunoreactivity in the dentate gyrus decreases as the animal ages. This may be explained by the disappearance of PS immunoreactivity from the nuclei after P14, the relative decrease in the cytoplasm of mature granule cells with expanded nuclei such that weaker immunoreactivity occurs because PS mainly exists in the cytoplasm, or because PS immunoreactivity is stronger in immature granule cell cytoplasm than mature granule cells (Fig. 8*a,b*). In fact, PS immunoreactivity in the cytoplasm and nuclei in these younger neurons was strong (Figs. 2*a,b, 2a,b* *,* *5*).

Neuronal differentiation in the dentate gyrus was not complete at P7, and PS was synthesized in immature granule cells at this time (Figs. 2*b*, 3*b*). After P14, PS was expressed at low levels in immature (PSA-NCAM-positive, NeuN-negative) granule cells (Figs. 2*k*, 3*k*), but mature granule cells and interneurons had strong PS immunoreactivity (Figs. 2*l*, 3*l*). Thus, immature neurons in the dentate gyrus of young animals highly express PS, but those in adult animals do not express PS. Furthermore, many immature neurons at P3 and P7 contained PS in their nuclei (Figs. 2*a,b*, 3*a,b*, 8*a*), and no PS expression was observed in the nuclei of mature or immature neurons in the adult animals. PS plays a significant role in development via its involvement in signal transduction pathways, cell proliferation, and apoptosis in cells of non-neurological origin [26]. Sun et al. reported that exogenous PS is predominantly localized in the nucleus of cultured cells [42], and our results show that endogenous PS also enters the nucleus under certain conditions.

PSA-NCAM-positive cells predominantly exist in the SGZ, the basal side of the granule cell layer of the dentate gyrus, but PS-positive cells localize to the apical side of the granule cell layer at P28. This study shows that most PS-positive cells in the dentate gyrus at P28 are NeuN-positive mature neurons. Double-positive neurons were rarely observed in the SGZ at P28. PSA-NCAM-positive cells in the SGZ extend their dendrites through the granule cell layer composed of many PS-positive neurons (Figs. 2*k,l*, 8*d*).

Yokota et al. and Hiraiwa et al. reported a significant increase in PS mRNA after a transient focal cerebral ischemia [16], [49], suggesting a pivotal role for PS in the survival of injured neurons. The PS isoforms Pro+9 and Pro+0 are caused by differential splicing at a splicing site in the saposin B domain [17]. Pro+9 has a 9-base (CAG GAT CAG: Gln Asp Gln) insertion whereas Pro+0 does not. Pro+0 is mainly transported into lysosomes, but Pro+9 is predominantly secreted out of a cell [25]. In addition, their expression pattern is specific in humans, mice, and rats [16], [51]. Pro+0 is mainly expressed in the lung, kidney, and liver, whereas Pro+9 is expressed in the brain, and skeletal and cardiac muscle [51]. The ratio of the two is typically 85∶15 of Pro+9:Pro+0; however, after brain injury, the ratio changes to 95∶5, and Pro+9 was reported to maintain expression throughout the injury [7], [16]. Pro+9 in the brains of chicken and mice increases during process of embryonic development [5]. However, the exact relationship between Pro+9 and the brain remains unknown.

Here, we analyzed the expression of both types of PS mRNA in the dentate gyrus after birth. The expression pattern of Pro+0 mRNA at P7, P14, and P21 was similar, but expression of Pro+9 mRNA independently increased at P21 (Fig. 6*i*). Pro+9, the secretion type of PS, is produced by mature granule cells but not by immature cells in the SGZ (Fig. 6*g–i*). These results suggest that PS is secreted in a paracrine manner from mature granule cells toward developing neural stem cells in the SGZ and plays an important role in neuronal proliferation or differentiation, such as dendrite expansion (Fig. 8*c,d*). Indeed, administration of PS or a small peptide mimicking the neurotrophic activity domain of PS is reported to facilitate neurite outgrowth [3] as well as prevent apoptosis [12], [21], [35].

At P28, saposin B immunoreactivity was detected in a number of PCA-NCAM-positive cells in which PS immunoreactivity was rarely observed (Fig. 5). Furthermore, PS mRNA expression was minimal in the SGZ in rats at similar stages (Figs. 6, 7). These results suggested that PSA-NCAM-positive immature neurons in the SGZ uptake PS by endocytosis and degrade PS into saposins A–D. By this mechanism, PS might stimulate the growth of dendrites and axons [30], [48], thereby influencing network formation. However, antibodies and hybridization probes are inherently different, and so these results must be interpreted carefully.

PS is both a precursor of sphingolipid hydrolase activators and a neurotrophic factor. In fact, a 12-amino acid neurotrophic activity domain was identified in the N-terminal portion of saposin C [30]. The neurotrophic activities of the peptide encompassing this domain have been demonstrated *in vivo* and *in vitro* [12], [21], [30], [35], [44]. However, the alternate splicing site of PS is located in the saposin B domain, which generates different forms of PS. Lamontagne and Potier reported that saposin B derived from both Pro+9 mRNA and Pro+0 mRNA differed in binding affinity for G_M1_ ganglioside, sulfatide, and sphingomyelin [23]. However, subsequent research found no significant functional differences in terms of lysosomal hydrolase activation between these saposin B isoforms [14]. Thus, the functional significance of PS isoforms remains unclear, but the tissue-specific expression of each mRNA type has been documented. We observed expression of both alternative splicing forms in the dentate gyrus with increased expression of Pro+9 mRNA in the granule cell layer and constant expression of Pro+0 mRNA, suggesting that the saposin B domain plays an important role in development of nervous tissue, and that proliferation and differentiation are regulated by both splicing forms of PS mRNA. These results also imply that PS has diverse neurotrophic functions in neurogenesis in young or adult animals.

Neurons in the brain are not thought to regenerate if they are injured. Many studies associated with neurogenesis are being undertaken worldwide in an effort to challenge this established theory and overcome serious clinical effects. PS has neurotrophic activity and may be related to neurogenesis. The dentate gyrus is part of the hippocampus that is involved in memory and learning; therefore, neurogenesis in the dentate gyrus is thought to be related to memory. This study may provide data regarding treatment for diseases related with memory loss.

## Supporting Information

Checklist S1 The ARRIVE Guidelines Checklist.
(DOC)
